# Optical Fiber Sensing-Aided 3D-Printed Replacement Parts for Enhancing the Sensing Ability of Architectural Heritage

**DOI:** 10.3390/mi14122135

**Published:** 2023-11-22

**Authors:** Weile Jiang, Kun Yao, Qijing Lin, Yulong Zhao, Di Lu

**Affiliations:** 1 School of Humanities and Social Sciences, Xi’an Jiaotong University, Xi’an 710049, China; 2 School of Mechanical Engineering, Xi’an Jiaotong University, Xi’an 710049, China; 3 School of Mechanical and Manufacturing Engineering, Xiamen Institute of Technology, Xiamen 361021, China; 4 Shandong Laboratory of Advanced Materials and Green Manufacturing at Yantai, Yantai 264003, China; 5 Bright Stone Industrial Technology Research Institute, Yantai 265503, China; 6 Institute of Modern Technology and Conservation of Cultural Heritage, Xi’an Jiaotong University, Xi’an 710049, China

**Keywords:** architectural heritage protection, 3D printing replacement parts, optical fiber sensing, temperature, strain

## Abstract

This study discussed the application of optical fibers in addressing the problem of insufficient light harvesting and sensing health monitoring in ancient buildings. Based on three-dimensional (3D) printing technology to fix the light-harvesting lens and conducting optical fiber, develop the replacement parts that can be buried into the optical fiber of ancient buildings. By introducing the experimental application to improve the experimental quality of research and teaching. Firstly, it highlights the advantage that the optical fiber plus lens structure design can make the natural light introduced for a long time; secondly, it points out that the buried optical fiber structure design does not affect the warmth and sound insulation of the building; finally, the health monitoring of the building is realized through the proposed method of buried optical fiber sensing. The design scheme adopts a fiber optic light transmission and sensing system, which can realize the whole system’s corrosion resistance, after laying buried and low-cost operation.

## 1. Introduction

**Figure 1:**
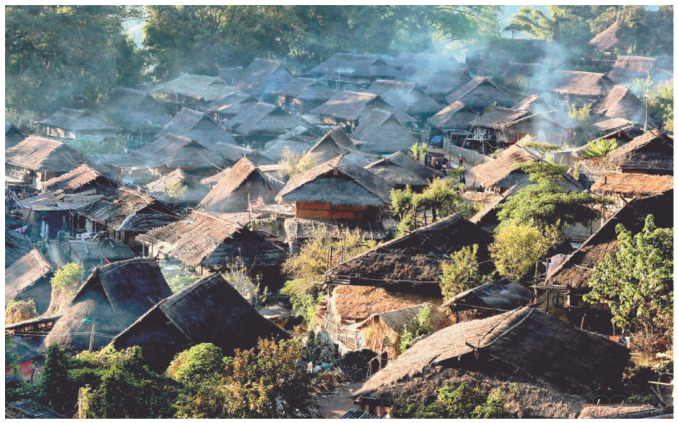
Wengding Village, the last primitive village in China [9].

Based on the Nara Document of Authenticity, large-scale replacement of builds or frames is also an isolated case for wood-framed architectural cultural heritage (e.g., Ise Jingu Shrine in Japan), and more often than not, it is a restorative replacement of localized and severely damaged elements. In recent years, the issue of environmental attributes and preventive conservation of architectural heritage has been increasingly emphasized [1,2,3]. The destruction of Notre Dame de Paris in 2019 and the Old Walled Village of Wengding Village in Yunnan Province in 2021 by fire have caused significant losses to human heritage. Located in Cangyuan Wa Autonomous County, Lincang City, Yunnan Province, Wengding Village has preserved the complete and ancient Wa folkways and customs, and is home to the Wa people who have inherited it for more than 4000 years, preserving the primitive residential architecture and its unique Wa culture, which is regarded as “the last primitive tribe in China”. However, due to the lack of sensor monitoring, the fire hazards were not detected in time. At 17:40 on 14 February 2021, a serious fire occurred in the old village of Wengding Village, and the last primitive tribe was burned down, as shown in Figure 1. To eliminate the hidden safety hazards in time, and to avoid the influence of the external environment on the ancient buildings and their damage, it is necessary to set up the necessary sensors for the ancient buildings to achieve the detection of the safety status. At present, the health detection of ancient buildings is mainly based on electric sensors, and the use of electric devices makes it easy to bring electric heat, electric sparks, and other safety hazards to the wooden structure, which can cause a fire. In order to address this problem, it is necessary to design a fiber optic measurement system without sparks, corrosion resistance, and oxidation resistance to monitor the safety of ancient buildings, which is small, implantable, and it is easy to integrate the advantages of fiber optic grating (FBG) sensing structure [4,5,6]. With the development of fiber optic sensing and 3D printing technology [7,8], based on 3D printing technology, the fiber optic can be directly buried into the restorative replacement parts of ancient buildings to enhance the reliability and stability of sensing.

In addition, ancient Chinese buildings, such as Buddhist temples and Taoist temples, often lacked effective lighting and ventilation systems, and the depth of the temple was large, resulting in a dark and humid environment that was not conducive to the protection of wooden structures. To improve the lighting environment inside the temple, the common ways include electric lighting or candlelight lighting methods, which will also bring fire risk to the wooden structure of the building. Natural light, as a valuable gift of nature to mankind, can be said to be an inexhaustible source of green energy. Compared with electric light sources, light-guided lighting technology based on natural light [10,11] is safer because it does not produce open flames. Photoconductive lighting technology is also characterized by clean energy, and photoconductive lighting systems have been used in some buildings in China, such as the Beijing Olympic Park Center and Changhong International City in Mianyang City, Sichuan Province. A structural health monitoring system based on optical fiber grating (FBG) has been developed by Jiang et al. [12] to monitor structural deformation and fire around the building for Chinese ancient buildings. Bai et al. [13] have addressed a model of strain and temperature measured for a typical Tibetan ancient building using 8 FBG sensors installed on the structure. It can be seen that FBG-based sensors are widely used in ancient building inspection.

This study discussed the application of optical fibers in addressing the problem of insufficient light harvesting and sensing health monitoring in ancient buildings. A highly reliable, high-performance fiber optic sensing and fiber optic light conduction system for the monitoring of temperature and strain key state parameters of ancient buildings was designed, as well as the realization of natural light conduction and illumination, which realizes the protection of ancient cultural heritage and has important theoretical research significance, engineering application value, and market application potential.

## 2. Materials and Methods

A fiber-optic buried light-inducing and sensing system with 3D printing technology is proposed aiming at the repair and replacement of ancient architectural loss structures. Multi-beam light through the lens focusing is introduced into the room. The addition of carbon fibers in polyethylene plastic can increase the toughness of the 3D printing material. The coefficient of thermal expansion and mechanical toughness of the doped carbon fiber composite materials are more similar to the wood, which makes it more suitable for partial replacement of the wooden structure. The light-harvesting area of the light-guided lighting system is based on the principles of transmission and refraction. The fiber optic bundle is placed at the focal point of the lens to efficiently collect sunlight and direct it into the interior of the fiber optic. Compared with the traditional skylight and light panel, the light guide lighting system has a small opening area, and the light entering the room is very uniform and soft. The light is in the full spectrum, and can effectively isolate more than 99% of the ultraviolet. The device does not need to be repeatedly cleaned, only the usual wind and rain can achieve a self-cleaning function. The whole system is fully sealed and in a semi-vacuum state, so it has a good thermal insulation effect and avoids the entry of dust and flying insects. Some parts of the ancient building are replaced with polyethylene composite materials embedded in optical fiber, which on the one hand realizes the indoor reference of sunlight by the light guide system, and on the other hand realizes the health monitoring of the temperature and structural deformation of the ancient building.

In the structural design, the lighting lens and conduction fiber are fixed based on 3D printing technology, and multiple fibers are introduced at the focal point of the lens. The replacement parts that can be embedded in fiber optic composite materials are made and installed in the defects of ancient buildings. Natural light can be introduced for a long time through the design of fiber plus lens structure. The temperature and stress data detected by distributed fiber grating were analyzed to realize the health monitoring of ancient architectural objects. The design scheme uses a fiber optic system, which can achieve full system corrosion resistance and low-cost operation after laying.

The printing material is polyethylene doped with carbon fiber. Polyethylene resin is an odorless, non-toxic white powder or particle. Its appearance is milky white, with a waxy feel. Polyethylene has low water permeability and is suitable for making moisture-proof structures. The low tensile strength of polyethylene leads to its poor creep resistance, so it is necessary to add carbon fiber to enhance the mechanical properties. In addition, the addition of carbon fiber to polyethylene plastic can make the thermal expansion coefficient and mechanical toughness of the printing material more similar to wood and more suitable for partial replacement of wooden structures.

The influence mechanism of laying design on fiber performance and the influence of additive manufacturing parameters on fiber binding force are still unclear. Therefore, the temperature and strain test platform has been set up to verify the temperature and stress characteristics of the optical fiber after embedding. The adhesion between optical fiber sensor and printing material is analyzed. The performance analysis and application research of optical fiber transmission and sensing are carried out.

## 3. Results

For the introduction of sunlight and health monitoring of ancient buildings and caves, the system is designed with a combined Fresnel lens, using sensors, mechanical devices, temperature and strain sensing, and demodulation modules to collect sunlight, apply the principle of spectroscopy, eliminate the unfavorable components of sunlight, and then use fiber optic couplers to import them into optical fibers, and then realize indoor illumination and sensing of ancient buildings after a long-distance transmission. The use of a light-harvesting cover realizes the efficient collection of sunlight and natural light, which is then fed into the interior of the system. The light-harvesting cover has an efficiency of over 90% in isolating ultraviolet rays, and at the same time has a smooth and translucent appearance, which can achieve a self-cleaning effect under the effect of wind and rain. In terms of system construction, based on 3D printing technology, we need to bury the optical fiber into ancient buildings to restore the location, build a fiber optic sensing scheme, and carry out photoconductive lighting performance testing.

### 3.1. Optical Guidance System Design

According to the way of lighting technology, they are divided into active and passive two kinds. Active concentrators are quite expensive to build. Therefore, the most used at present is the passive lighting guide. The light cover and light conduit are linked together and fixed. According to the characteristics of the building structure, the light conduit is processed and installed in a fixed size. The condenser is mostly made of glass injection molding, and the surface has a triangular full-reflection condenser edge. The installation method of the light guide tube is divided into top lighting and side lighting. At present, most of the buildings use top lighting. In this paper, passive roof lighting is adopted.

**Figure 2:**
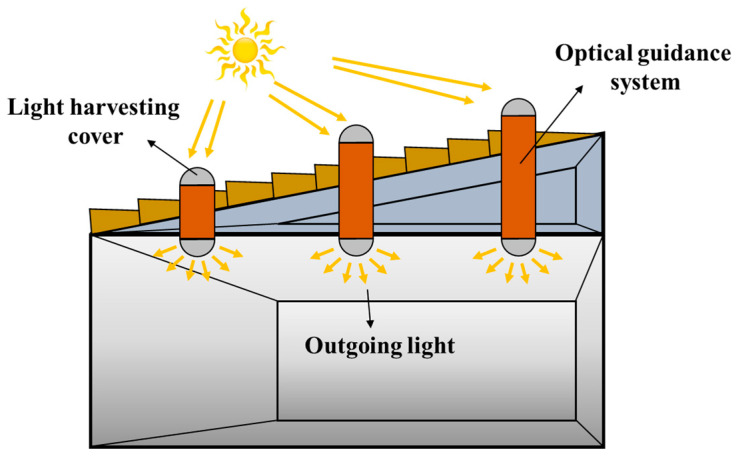
The design scheme of the photoconductive system.

As shown in Figure 2, the entire optical guide system is composed of a daylighting cover, transmission fiber, and output fiber. The transmission fiber is selected as a large numerical aperture and low loss fiber, which can transmit light at least 20 m, and can also make any turn within 90°, ensuring a wider transmission range and higher transmission efficiency. The use of diffusers in the output area can be more uniformly concentrated natural light, large area dispersed irradiation to every corner of the indoor lighting needs, whether it is early in the morning, late in the evening, or cloudy and rainy weather, through the light pipe lighting system to the indoor introduction of light can ensure that the indoor light is sufficient. Diffusers usually use a 3D nano diffusion lens or acrylic, which has high hardness. They have good transmission (>90%) and diffusion ability. The light after diffuse treatment is full spectrum, with no glare and no stroboscopic light, and the light can be adjusted and controlled by the dimmer, so as to better fit the lighting needs of the room.

### 3.2. Burial Process for Fiber Optic Sensors

**Figure 3:**
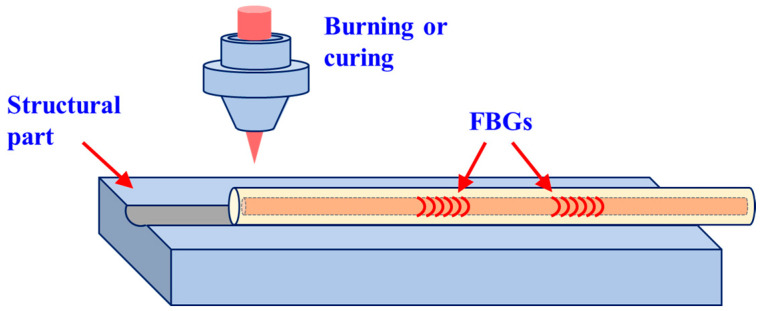
Fiber-embedded 3D printing site.

Pure quartz fiber grating based on femtosecond laser inscription is used as a sensing probe to explore the influence of the preparation process and structural design on the grating structural stability, optical performance, and response sensitivity. Plastic material doped with carbon fiber is used as raw material, as shown in Figure 3, to explore the tolerance of buried fiber optic sensors in complex environments. Based on plastic 3D printing technology, this paper designed the optical fiber embedding scheme of ancient buildings, made trajectory planning for the landfill position of optical fiber sensors, and carried out an error analysis between the 3D printing model and the ancient building restoration.

### 3.3. Design of Experiments for Sensing and Optical Guidance

#### 3.3.1. Temperature and Strain Sensing Test

Fiber-optic sensors are capable of measuring a variety of covariates [5,14,15]. For some complex systems such as civil engineering structures, temperature and strain do not correspond linearly [16,17]. Therefore, the measurement characteristics need to be calibration. A high-temperature muffle furnace and a strain bench were selected to provide a temperature and strain testing environment. The temperature and strain coefficients of the restored body of the ancient building with the buried sensors were tested for sensing respectively. The replacement parts with buried optical fibers were placed in the temperature control box for calibration and testing to determine the temperature characteristics of the fiber optic sensors. Then, strain calibration and tests of the sensor are carried out.

#### 3.3.2. Illuminance Test

Compared with the traditional lighting method, the non-electric light guide system lighting has the advantages of fire prevention, heat insulation, and uniform and soft light. In general, under cloudy conditions, for a set of 530 mm diameter light guide lighting system, when the lighting area is 80 m^2^, the average illuminance must reach the national standard of 75 LX. When the lighting area is about 40 m^2^, the illumination needs to reach 250 LX. Therefore, 80 m^2^ and 40 m^2^ darkroom space should be designed in the experiment to test the illuminance of the light guide system. Our team has cooperated with ancient building units in the early stages to install partial replacement parts for ancient buildings in need of restoration and verify sensing characteristics and illumination characteristics.

## 4. Conclusions

In this paper, through theoretical analysis combined with experimental investigation, we actively cross disciplines and focus on exploring the ways and means of integrating fiber-optic light-conducting and sensing integrated sensors. Fiber optic conduction and sensing systems have the advantages of anti-electromagnetic interference, high sensitivity, compactness, chemical corrosion, and oxidation resistance. Based on the design of buried fiber optic sensors and light intensity loss analysis, a light guiding system for the area of 80 m^2^ and 40 m^2^ is proposed to achieve the effective illuminance of the national standard. Temperature and strain sensing characteristics of the designed sensor are tested. The temperature measurement range is from −10 to 100 °C with an accuracy of ±1%. The strain measurement range is from 0 to 4 MPa with an accuracy of ±1%. Based on the buried optical fiber laying technology, the fiber optic conduction and sensing technology is applied to the optical conduction and health monitoring of the cultural heritage, which can make up for the deficiency of the traditional electric measuring instruments, promoting the process of fiber optic conduction and sensing, improving the experimental quality of research and teaching, and can, to a certain extent, contribute to the improvement of the standard of living of the people.

## Data Availability

Data and materials can be obtained by contacting the corresponding author.
